# Genome-Wide Association Study of Autistic-Like Traits in a General Population Study of Young Adults

**DOI:** 10.3389/fnhum.2013.00658

**Published:** 2013-10-11

**Authors:** Rachel Maree Jones, Gemma Cadby, Phillip E. Melton, Lawrence J. Abraham, Andrew J. Whitehouse, Eric K. Moses

**Affiliations:** ^1^Centre for Genetic Origins of Health and Disease, University of Western Australia, Perth, WA, Australia; ^2^School of Chemistry and Biochemistry, University of Western Australia, Perth, WA, Australia; ^3^Telethon Institute for Child Health Research, Perth, WA, Australia

**Keywords:** autistic-like traits, genome-wide association, PRKCB1, autism spectrum disorder, autism spectrum quotient, CBLN1

## Abstract

**Lay abstract:** It has been proposed that autistic-like traits in the general population lie on a continuum, with clinical Autism Spectrum Disorder (ASD), representing the extreme end of this distribution. The current study undertook a genome-wide association (GWA) scan of 965 young Western Australian adults to identify novel risk variants associated with autistic-like traits. No associations reached genome-wide significance; however, a review of nominally associated single nucleotide polymorphisms (SNPs) indicated two positional candidate loci that have been previously implicated in autistic-like trait etiology.

**Scientific abstract:** Research has proposed that autistic-like traits in the general population lie on a continuum, with clinical ASD representing the extreme end of this distribution. Inherent in this proposal is that biological mechanisms associated with clinical ASD may also underpin variation in autistic-like traits within the general population. A GWA study using 2,462,046 SNPs was undertaken for ASD in 965 individuals from the Western Australian Pregnancy Cohort (Raine) Study. No SNP associations reached genome-wide significance (*p* < 5.0 × 10^−8^). However, investigations into nominal observed SNP associations (*p* < 1.0 × 10^−5^) add support to two positional candidate genes previously implicated in ASD etiology, PRKCB1, and CBLN1. The rs198198 SNP (*p* = 9.587 × 10^−6^), is located within an intron of the protein kinase C, beta 1 (PRKCB1) gene on chromosome 16p11. The PRKCB1 gene has been previously reported in linkage and association studies for ASD, and its mRNA expression has been shown to be significantly down regulated in ASD cases compared with controls. The rs16946931 SNP (*p* = 1.78 × 10^−6^) is located in a region flanking the Cerebellin 1 (CBLN1) gene on chromosome 16q12.1. The CBLN1 gene is involved with synaptogenesis and is part of a gene family previously implicated in ASD. This GWA study is only the second to examine SNPs associated with autistic-like traits in the general population, and provides evidence to support roles for the PRKCB1 and CBLN1 genes in risk of clinical ASD.

## Introduction

Autism Spectrum Disorders (ASD) represent a group of neurodevelopmental conditions characterized by impairments in social interaction and communication, and repetitive interests and behaviors. A recent review estimated the median global prevalence of ASD at 62 cases per 10,000 children (Elsabbagh et al., 2012). The overall heritability of ASD is estimated at 90%, which is amongst the highest for any neuropsychiatric disorder (Hollander et al., 2003; Skuse et al., 2005). Evidence suggests that ASD have a complex inheritance where different, likely overlapping, groups of genetic variants may cause susceptibility to disease (Veenstra-VanderWeele et al., 2004). Increasing numbers of epidemiological and genetic studies have deepened the understanding of genetic contribution to ASD, and show that a variety of genetic mechanisms may be involved in the etiology (Li et al., 2012).

Autistic-like traits are sub-threshold deficits in socialization, communication, and restricted interests that do not meet formal criteria for ASD (Constantino and Todd, 2003). Several authors have suggested that ASD can be conceptualized as the conditions arising in individuals found at the extreme end of a normal distribution of autistic-like traits (Gillberg, 1992; Constantino and Todd, 2003; Ronald et al., 2005). Population-based studies have supported this by finding that, in addition to individuals with ASD, many others exhibit sub-threshold autistic or autistic-like traits (Constantino and Todd, 2003; Posserud et al., 2006; Lundström et al., 2012). Sub-threshold autistic-like traits have been examined in general population twin studies, with heritability estimates ranging from 36 to 87% in these studies (Ronald and Hoekstra, 2011).

With the discovery of common genetic variants associated with ASD, the question is raised whether common risk loci also contribute to variation in phenotypes such as autistic-like traits (St Pourcain et al., 2010). One methodological approach that is gaining influence in ASD research is the examination of the quantitative distribution of autistic-like traits in the broader community, rather than a dichotomy of ASD cases and controls. Many authors have postulated that common genetic variants that are present in a significant proportion of the general population may play a role in the etiology of ASD (Campbell et al., 2006; Alarcón et al., 2008; Chakrabarti et al., 2009; Wang et al., 2009; Anney et al., 2010; Ronald et al., 2010). Further to this, it is thought that understanding the etiology of individual differences in autistic-like traits in the general population will help in understanding the causes of ASD (Ronald and Hoekstra, 2011).

The aim of the current study was to use genome-wide association (GWA) methods to search for novel risk variants associated with autistic-like traits in a Western Australian population sample.

## Materials and Methods

### Ethics statement

Participant recruitment of the study families were approved by the Human Ethics Committee at King Edward Memorial Hospital. The 20 year follow-up ethics approval was received from the Human Research Ethics Committee at the University of Western Australia. Participants provided written informed consent for data collection of autistic-like trait outcomes at approximately 20 years of age.

### GWA study sample population

Data were obtained from the Western Australian Pregnancy Cohort (Raine) Study based at the Telethon Institute for Child Health Research. The Raine Study is a longitudinal investigation of pregnant women and their offspring, who were recruited from King Edward Memorial Hospital, Perth, Western Australia or nearby private practices between 1989 and 1991 (Newnham et al., 1993). From the 2,900 pregnancies recruited into the Raine Study, 2,868 live-born children have been followed since the commencement of the study. The final sample consisted of 965 individuals from the Raine study with both genotype and outcome measures.

### Measure of autistic-like traits

At the 20 year follow-up, Raine Study participants who did not have a diagnosis of intellectual disability or ASD were asked to complete the Autism Spectrum Quotient (AQ) (Baron-Cohen et al., 2001). The AQ is a self-report questionnaire that provides a quantitative measure of autistic-like traits in the general population (Baron-Cohen et al., 2001). Individuals are provided with 50 statements and asked to indicate on a 4-point scale how well that statement applies to them (strongly agree, agree, disagree, strongly disagree). The items were scored on a scale ranging from 1 to 4, based on previous research that this scoring method retains more information about responses than the dichotomous scoring first proposed for this instrument (Baron-Cohen et al., 2001; Austin, 2005; Stewart and Austin, 2009; Russell-Smith et al., 2011). Scores on each item are summed to provide a Total AQ, with higher scores indicating greater autistic-like traits. The Total AQ is known to have good test-retest reliability (*r* = 0.7), and validation studies have found that scores in the general population follow a normal quantitative distribution (Baron-Cohen et al., 2001; Whitehouse et al., 2011). Factor analyses of the AQ in several countries have consistently identified three clear factors, relating to social ability, attention to detail/patterns, and the understanding of others. In the current study, we divided items into the subscales identified in a study of Western Australian adults (Russell-Smith et al., 2011), who were highly similar to the sample under investigation here: Social Skills, Details/Patterns, and Communication/Mindreading. There is minimal difference between the items pertaining to these subscales, and those reported in other factor analyses. For the current data set, internal reliability for the scales ranged from moderate (Communication/Mindreading: α = 0.63) to good (Details/Patterns: α = 0.78) and excellent (Social Skills: α = 0.85). In this study, Total AQ scores and the scores of the three subscales were used as four outcome measures.

### GWA study genotyping

**Figure 1:**
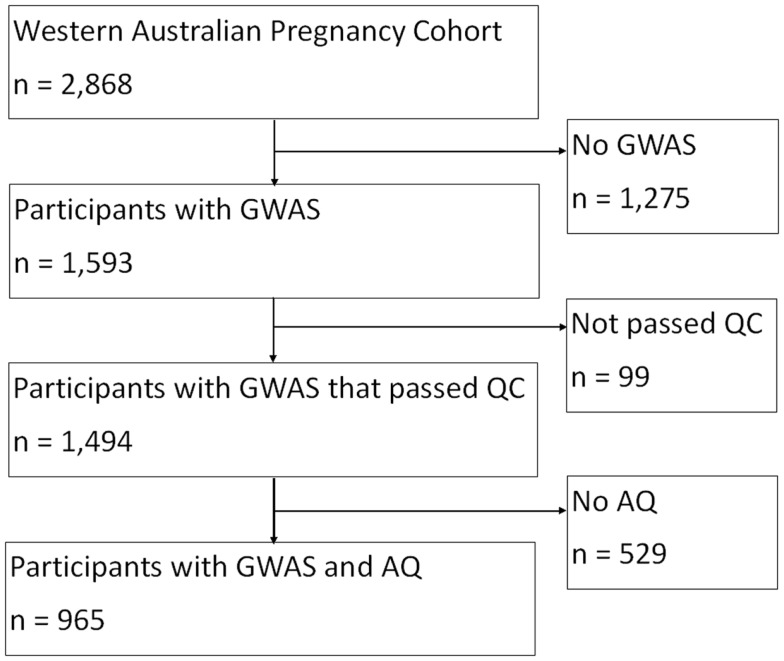
**Flow chart of study sample selection**.

In the Raine Study, DNA was collected using standardized procedures from 74% of adolescents who attended the 14 year follow-up, and a further 5% at the 16 year follow-up. Of the 1,593 offspring for whom genome-wide data were available, 99 individuals were excluded for the following reasons: 16 had low genotyping success ( >3% missing); 3 had excessive heterozygosity, 68 were in high identical-by-descent with another Raine Study participant (related), 7 whose sex was ambiguous, and 5 individuals had mislabeled samples. There were 1,494 individuals whose DNA sample passed quality control criteria and were eligible for the genetic analyses (768 males, 726 females), see Figure 1.

Genome-wide data were generated using an Illumina Human 660W Quad array at the Center for Applied Genomics (Toronto, ON, Canada). The 660W Quad Array includes 657,366 genetic variants including ∼560,000 single nucleotide polymorphisms (SNPs) and ∼95,000 copy number variants. SNPs were excluded based on the Wellcome Trust Case Control Consortium thresholds:

HWE *p-*value must be >5.7 × 10^−7^ (919 markers excluded)
Call rate must be >0.95 (95%) (97,718 markers excluded)
MAF must be >0.01 (1%) (119,246 markers excluded – includes copy number variants)
A/T and G/C SNPs were removed due to possible strand ambiguity.

In total, 535,632 SNPs passed genotype quality control before genotype imputation. Imputation was performed using MACH (Li and Abecasis, 2006) software on the 22 autosomes. Once the data were cleaned, a two-step process was carried out using the CEU samples from HapMap phase2 build 36 release 22 as a reference panel. This imputation process imputed both individual genotypes for SNPs that were typed but not called for a particular individual, and SNPs that were not typed in all individuals. After imputation, genotype information was available for 2,543,887 SNPs.

### Data analysis

#### Power calculations

Power calculations were performed using the statistical software package Quanto (Gauderman, 2002) for a sample size of 965 and MAF ranging from 0 to 0.5. The means and standard deviations for Total AQ and each subscale were used for the power calculations. Power was calculated to detect a minimum change of one unit assuming an additive genetic model. An alpha level of 1.0 × 10^−8^ was used for the GWA studies.

#### Methods for genome-wide association

Allelic dosage scores generated from MACH were used in analyses. The GWA studies were run adjusting for both age and sex. To account for potential population substructure, first and second principal components were also included in the multivariate GWA model. According to the Eigenvectors the first principal component accounted for 6.5% of variation, and the second component accounted for 2.3% of variation. Analysis was run using the ProbABEL package (Aulchenko et al., 2010). The combined results and SNP details file were then filtered to remove SNPs with MAF <1% and an imputation quality value of <0.3 (suggesting poor imputation quality).

A quantile–quantile (Q–Q) plot depicting –log10 transformations of the observed *p-*values as a function of the expected *p-*values was generated using “*qqunif*” function of the R Genetic Analysis Package (GAP) (R Development Core Team, 2008). The genomic inflation factor, λ, was calculated from the observed *p-*values. A Manhattan plot displaying a –log10 transformation of the observed *p-*values was generated using “*mhtplot*” function of the R GAP (R Development Core Team, 2008).

Genome-wide significance of results was defined as a *p-*value ≤ 5.0 × 10^−8^ (Panagiotou and Ioannidis, 2011). Tables of SNPs were generated, based on a *p-*value < 1.0 × 10^−5^, which has been used by the National Human Genome Research Institute for the identification and archiving of suggestive associations (National Human Genome Research Institute, 2012). Information regarding genes and function for SNPs included in these tables were sourced from SNP Annotation and Proxy Search (SNAP, 2008). Genes and/or regions that appeared in the generated tables were searched using the Simon’s Foundation Autism Research Initiative (SFARI) database (Simons Foundation Autism Research Initiative, 2012) for any previous associations with ASD and biological plausibility. LocusZoom (Pruim et al., 2010) was used to generate a regional association plot for loci of interest (±400 kb) based on hg18 genome build and HapMap Phase II CEU133 as the linkage disequilibrium (LD) population.

## Results

### Power calculations

Total AQ, Social Skills, and Details/Patterns did not reach sufficient power ( >80%) to detect a change by one unit. Communication/Mindreading reached sufficient power at 47% MAF.

### Normal distribution of scores

Autism Spectrum Quotient scores have been described in previous literature to provide a quantitative measurement of autistic-like traits in the general population (Baron-Cohen et al., 2001). Histograms and Q–Q plots indicate that the scores of the total AQ and each subscale were normally distributed. The histograms and Q–Q plots can be found in Figures S1–S4 in Supplementary Material.

### Descriptive statistics

Characteristics of the current study sample can be found in Table 1. About 51.3% of the sample were female and the mean age at AQ completion was 19.68 (SD = 0.70). Mean total AQ score was 103.2 (SD = 12.60).

**Table 1:** **Characteristics of Raine study participants with AQ and GWA data available**.

Continuous	*n*	Mean (SD)
Maternal age at conception	965	28.66 (5.65)
Paternal age at conception	646	31.68 (6.18)
Maternal BMI at conception	956	22.25 (4.00)
Paternal BMI at conception	827	24.44 (3.28)
Age at AQ completion	965	19.68 (0.70)
Total AQ score	965	103.2 (12.60)

**Categorical**	***n***	***n* (%)**

Season of birth	965	
Summer		301 (31.2)
Autumn		222 (23.0)
Winter		196 (20.3)
Spring		246 (25.5)
Sex	965	
Male		470 (48.7)
Female		495 (51.3)
Gestational age	955	
<32 weeks		13 (1.4)
32–37 weeks		157 (16.4)
38–40 weeks		624 (65.3)
>40 weeks		161 (16.9)
Family income	937	
Income < $24 k		293 (31.3)
Income > $24 k		644 (68.7)
Maternal education	956	
Secondary school not completed		501 (52.4)
Secondary school completed		455 (47.6)

### Genome-wide association results

After filtering for effect allele frequencies ≤1 and ≥99% and imputation quality *R* squared values <0.3, 2,462,046 successfully genotyped or imputed SNPs remained for analysis. No SNP associations in any outcome reached genome-wide significance, defined as *p-*value < 5.0 × 10^−8^ (Panagiotou and Ioannidis, 2011). Putative associations, defined as *p* < 1.0 × 10^−5^, were examined with respect to candidate genes in the SFARI Gene database. None of the genes that were putatively associated with Total AQ, Details/Patterns and Communication/Mindreading were found to be represented in this gene database. One putative gene association found in the Social Skills GWA results was found to be reported in the SFARI Gene database, prompting further investigation.

#### Social skills genome-wide association results

**Figure 2:**
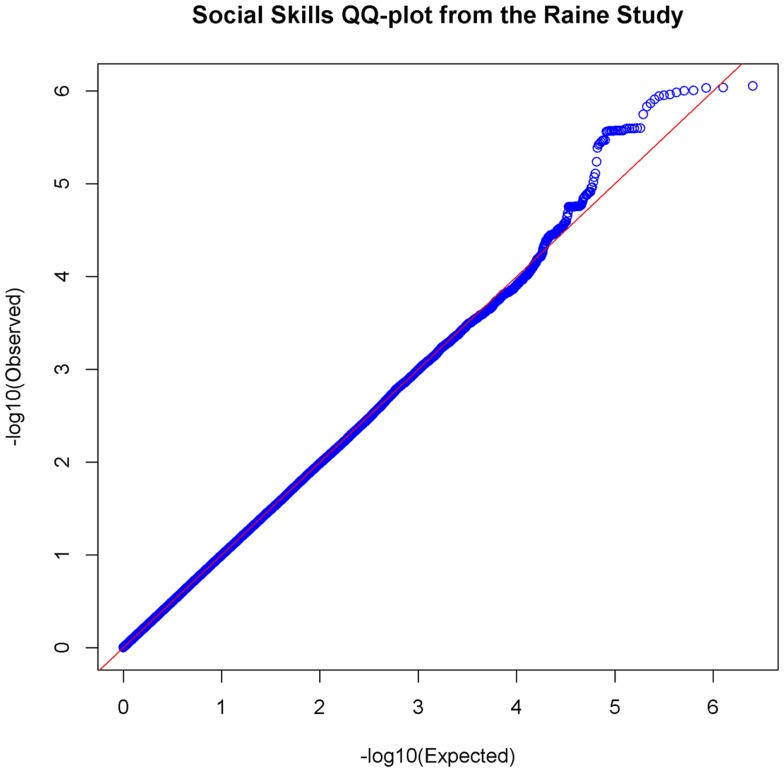
**Quantile–Quantile Plot of association results for social skills**. Q–Q plots compare the observed *p-*values [−log10(Observed)] to expected *p-*values. [−log10(Expected)] on the logarithmic scale under the null hypothesis of no significant association.

**Figure 3:**
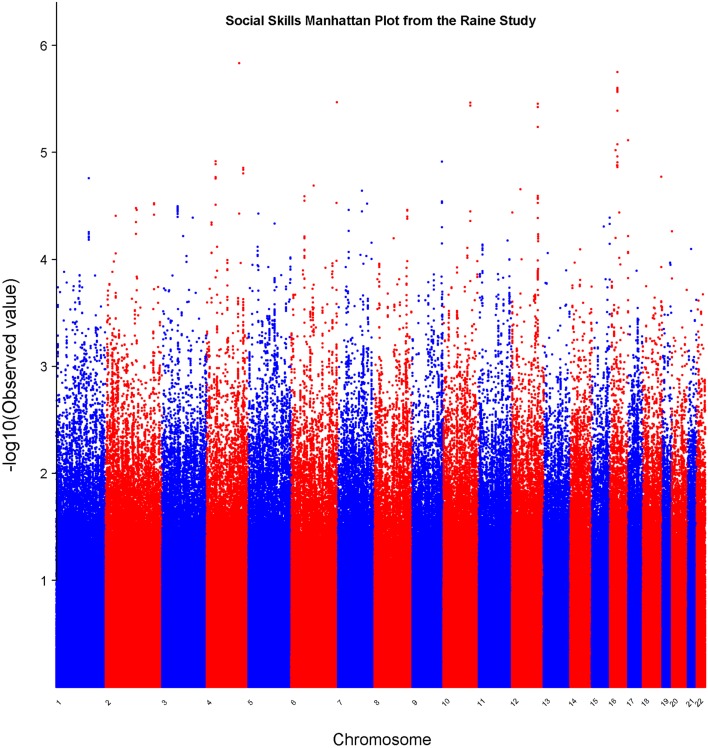
**Manhattan plot for social skills**. Statistical significance of each SNP on the −log10 scale as a function of the chromosome position.

In the set of 965 Raine Study participants for Social Skills scores, the observed distribution of *p-*values for the SNPs exhibited minimal deviation from the expected distribution (see Figure 2) indicating minimal test statistic bias or underlying population structure (λ = 0.994). The Manhattan plot –log10 transformation of observed *p-*values across the genome are displayed in Figure 3.

Table 2 displays association results for Social Skills, selected on a *p-*value below 1.0 × 10^−5^. None of these associations reached genome-wide significance. However, the rs198198 SNP (*p-*value = 9.587 × 10^−6^, effect size = −1.411) is located within the *protein kinase C*, *beta 1* (*PRKCB1*) gene that has been previously reported to be associated with ASD. Exploratory analysis of data revealed a nominal association of rs198198 with Total AQ score (*p-*value = 0.002), but no association with Details/Patterns (*p-*value = 0.434) or Communication/Mindreading (*p-*value = 0.202). In addition, the rs16946931 SNP (*p* = 1.78 × 10^−6^, effect size = 1.681) is located in a region flanking the *Cerebellin 1* (*CBLN1*), a gene previously implicated in ASD. This SNP was nominally associated with the Total AQ score (*p*-value = 4.27 × 10^−4^), but no association with Details/Patterns (*p*-value = 0.472), or Communication/Mindreading (*p*-value = 0.138). When association analysis was done conditional on rs16946931 for Social Skills, *p*-values for the 20 additional *CBLN1* SNPs became insignificant (*p*-value > 0.05), demonstrating that high LD between these SNPs was responsible for their inclusion in the model.

**Table 2:** **Association results for social skills (*p-*value < 1.0 × 10^−^^5^)**.

Chr	SNP	Position	Imputation	Function	Gene	Alleles	*R*^2^	Effect size	SE	*p-*Value
4	rs11947645	151321122	imp	Intronic	*DCLK2*	A/G	0.859	5.329	1.100	1.47E-06
16	rs16946931	47700007	imp	Intergenic	*N4BP1/CBLN1*	C/T	0.973	1.681	0.350	1.78E-06
16	rs7499215	47680431	gen	Intergenic	*N4BP1/CBLN1*	G/T	1	−1.639	0.346	2.51E-06
16	rs11860027	47679816	imp	Intergenic	*N4BP1/CBLN1*	C/T	1	−1.639	0.346	2.52E-06
16	rs16946881	47677288	imp	Intergenic	*N4BP1/CBLN1*	C/T	0.999	−1.639	0.346	2.53E-06
16	rs16946880	47676950	imp	Intergenic	*N4BP1/CBLN1*	C/T	0.999	−1.639	0.346	2.53E-06
16	rs16946876	47675600	imp	Intergenic	*N4BP1/CBLN1*	C/T	0.998	1.638	0.346	2.54E-06
16	rs9635530	47674912	imp	Intergenic	*N4BP1/CBLN1*	A/C	0.998	−1.638	0.346	2.54E-06
16	rs2883805	47693566	imp	Intergenic	*N4BP1/CBLN1*	G/T	0.995	−1.640	0.347	2.59E-06
16	rs4785161	47686589	imp	Intergenic	*N4BP1/CBLN1*	A/C	0.997	1.636	0.346	2.66E-06
16	rs753858	47685051	imp	Intergenic	*N4BP1/CBLN1*	C/T	0.998	1.635	0.346	2.66E-06
16	rs11859884	47670664	imp	Intergenic	*N4BP1/CBLN1*	A/G	0.995	−1.633	0.346	2.67E-06
16	rs1861572	47684489	imp	Intergenic	*N4BP1/CBLN1*	A/T	0.999	1.635	0.346	2.67E-06
16	rs1009302	47670836	imp	Intergenic	*N4BP1/CBLN1*	C/T	0.995	−1.633	0.346	2.68E-06
16	rs1009301	47670978	imp	Intergenic	*N4BP1/CBLN1*	G/T	0.995	1.633	0.346	2.68E-06
16	rs1345404	47681670	imp	Intergenic	*N4BP1/CBLN1*	A/G	1	−1.633	0.346	2.69E-06
16	rs1345406	47681539	gen	Intergenic	*N4BP1/CBLN1*	A/C	1	−1.633	0.346	2.69E-06
16	rs1345405	47681629	gen	Intergenic	*N4BP1/CBLN1*	A/G	1	−1.633	0.346	2.69E-06
16	rs1420612	47672854	imp	Intergenic	*N4BP1/CBLN1*	G/T	0.996	−1.631	0.346	2.72E-06
16	rs10521175	47673463	imp	Intergenic	*N4BP1/CBLN1*	A/C	0.997	1.629	0.345	2.75E-06
6	rs11575088	167477024	imp	Intergenic	*CCR6/GPR31*	A/C	0.998	1.620	0.347	3.40E-06
6	rs11575089	167477189	gen	Intergenic	*CCR6/GPR31*	A/G	0.999	1.620	0.347	3.41E-06
10	rs927821	104197789	gen	Intergenic	*MIR146B/LOC100505761*	A/C	0.985	1.7024	0.365	3.44E-06
12	rs10444533	105753835	gen	Intronic	*RIC8B*	C/T	0.996	1.458	0.313	3.54E-06
10	rs7086205	104192719	gen	Intergenic	*MIR146B/LOC100505761*	C/T	0.998	−1.688	0.362	3.67E-06
12	rs10778511	105749461	imp	Intronic	*RIC8B*	A/T	0.993	1.454	0.313	3.80E-06
16	rs1362594	47682845	imp	Intergenic	*N4BP1/CBLN1*	C/G	0.943	−1.648	0.356	4.09E-06
12	rs4964491	105746645	imp	Intronic	*RIC8B*	A/G	0.985	−1.429	0.314	5.79E-06
16	rs533581	87494938	imp	Intronic	*CBFA2T3*	C/T	0.859	1.363	0.303	7.75E-06
16	rs4785276	47678880	imp	Intergenic	*N4BP1/CBLN1*	A/G	0.974	−1.529	0.341	8.46E-06
16	rs198198	24035898	imp	Intronic	*PRKCB1*	A/T	0.777	−1.411	0.317	9.59E-06

Position: base pair. Imputation: imputed (imp) or genotyped (gen). Gene: for intergenic SNPs, 5′/3′ flanking genes listed. Alleles: effect allele/non-effect allele. *R*^2^: imputation quality from MACH. Effect size: regression coefficient. SE, Standard Error.

**Figure 4:**
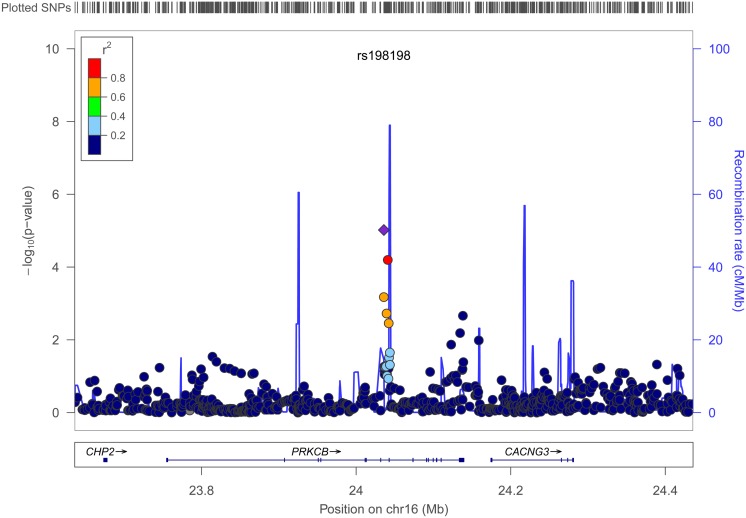
**Regional association plot of SNP rs198198 (±400 kb)**. Statistical significance of each SNP on the –log10 scale as a function of the chromosome position. The top SNP is shown as the purple diamond; the correlations (*r*^2^) of each of the surrounding SNPs to the top SNP are shown in the indicated colors. Recombination rate is shown in pale blue.

**Figure 5:**
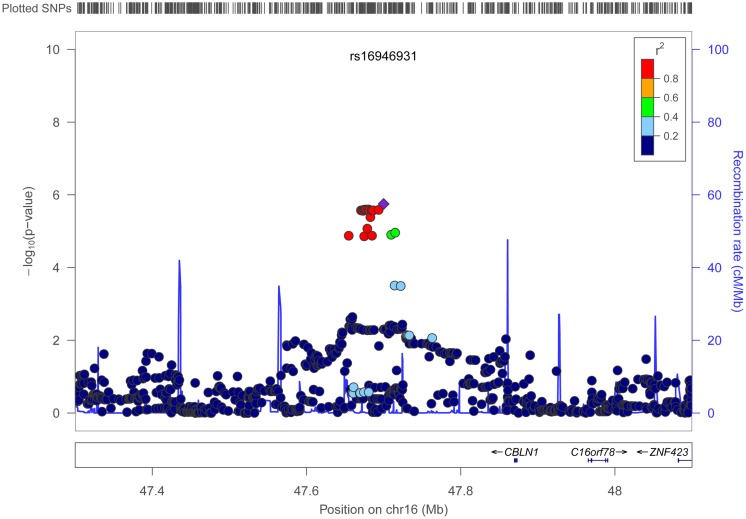
**Regional association plot of SNP rs16946931 (±400 kb)**. Statistical significance of each SNP on the –log10 scale as a function of the chromosome position. The top SNP is shown as the purple diamond; the correlations (*r*^2^) of each of the surrounding SNPs to the top SNP are shown in the indicated colors. Recombination rate is shown in pale blue.

Figures 4 and 5 show regional association plots of SNPs rs198198 and rs16946931, respectively.

### Bioinformatic evaluation

All SNPs with *p-*value below 1.0 × 10^−5^ were evaluated for possible functional potential. As all SNPs were in non-coding regions they were assessed for regulatory potential using available ENCODE data through Regulome DB (Boyle et al., 2012) and the UCSC Genome (2012) browser. No SNP was shown to be part of a transcription factor binding site or be located in a region of the genome likely to have regulatory potential except the nominally associated SNP rs198198 located within an intron of *PRKCB1*. The major allele (T) was found to be embedded within a near-consensus CCAAT/enhancer binding protein (C/EBP) gamma binding site whereas the minor allele (A) is predicted to ablate C/EBP gamma binding. Further evidence for function comes from ENCODE ChIP-seq data indicating that rs198198 is located in a region subject to histone H3K9 modification.

## Discussion

In this paper we report only the second contemporary GWAS for autistic-like traits in the broader community and the first to use a quantitative distribution measured by the AQ. Although we did not find any SNP associations reaching genome-wide significance (defined as *p-*value < 5.0 × 10^−8)^ this GWAS has provided some evidence to support the further investigation of two previously identified candidate genes, namely *PRKCB1* and *CBLN1*.

### *PRKCB1* gene

Protein kinase C enzymes play an important role in signal transduction, regulation of gene expression, and control of cell division and differentiation (Lintas et al., 2009; NCBI, 2012). The alternative splicing of *PRKCB1* generates two mRNA isoforms named PRKCB1-1 and PRKCB1-2, coding for two isoenzymes βI and βII. These mRNA isoforms are expressed in a variety of tissues within the central nervous system, including the hippocampus, striatum, suprachiasmatic nucleus, and cerebellar granule cells (Lintas et al., 2009).

Several previous genetic studies have supported a role for *PRKCB1* in ASD. A genome-wide linkage scan of 116 families from the Autism Genetic Resource Exchange cohort (Philippi et al., 2005) applied direct physical identity-by-descent mapping using a strict ASD phenotype. Their results demonstrated linkage to a region on chromosome 16p (*p-*value = 0.0027). Subsequent high-resolution SNP genotyping and analysis of this region showed that haplotypes in the *PRKCB1* gene were strongly associated with ASD (Philippi et al., 2005), a finding that was independently confirmed in two other family studies (Philippi et al., 2005) (Lintas et al., 2009) but not in a study on 171 Irish ASD trios (Yang et al., 2007). These haplotypes were not significantly associated with ASD in the current study (results not shown).

These genetic association studies have been supported by a study of PRKCB1 gene expression in post mortem brain tissue from 11 autistic patients and controls (Lintas et al., 2009). The superior temporal gyrus (BA 41/42) region was chosen for study because of a known role in processing socially relevant information and well documented structural and functional abnormalities seen in ASD (Zilbovicius et al., 2006). A significant down regulation of PRKCB1 gene expression was found to be associated with ASD (Lintas et al., 2009) providing additional strong evidence in support of a role for the *PRKCB1* gene in the etiology of ASD.

### *CBLN1* gene

The *CBLN1* gene encodes the cerebellum-specific precursor protein, precerebellin and is involved along with *Cerebellin 2* (*CBLN2*) in encoding ligands for the neurexin-neuroligin trans-synaptic complex (NTSC) (Clarke et al., 2012; Cristino et al., 2013). *CBLN1* belongs to the CBLN subfamily of the C1q/tumor necrosis factor superfamily, which plays a role in intercellular signaling, neuronal cell adhesion, brain development, and formation of synapses (Clarke et al., 2012). In a mouse study, Uemura et al. (2010) found that post-synaptic GluRδ2/GRID2 (a member of the δ-type glutamate receptor) interacts with pre-synaptic neurexins through *CBLN1* to mediate parallel fiber-Purkinje cell synaptic formation in the cerebellum. A recent study has identified rare copy number variants in NTSC gene family members and proposed their potential biological function with ASD and Tourette syndrome (TS) (Clarke et al., 2012) and mutations in these and other synaptic genes are common in ASD (Arons et al., 2012; Clarke et al., 2012). Therefore, it is plausible that long-range genetic variants affecting *CBLN1* expression may be associated with ASD traits.

While no previous genetic linkage or association studies have detected significant associations between *CBLN1* and ASD, *CBLN2* (belonging to the same CBLN subfamily) on Chromosome 18q has been deleted in TS and is located near two TS translocation breakpoints (Clarke et al., 2012). There is evidence to suggest an overlap between TS and ASD from epidemiological perspectives, with an increased prevalence of TS among autistic patients of 6.5% (Baron-Cohen et al., 1999), compared to between 2 and 3% in the general population (Mason et al., 1998). Common susceptibility genes (Sundaram et al., 2010; Fernandez et al., 2012) have also been reported between ASD and TS.

This study was conducted on a well characterized, population-based cohort of adolescents and ASD measurements were collected without ascertainment for autism risk. Despite this study’s small sample size (*n* = 965), we were independently able to identify two regions on chromosome 16 that have been previously implicated in the etiology of ASD. While no variants in these regions reached genome-wide significance, they do demonstrate suggestive evidence for association and provide good candidates for future follow-up studies. The difference in observed signal between the two regions is due to high LD around the *CBLN1* SNP, rs16946931, which disappears with conditional association analysis. The lack of replication for the previously identified *PRKCB1* haplotype is potentially due to different underlying LD structure between populations, as the previous study focused on families (Philippi et al., 2005) whereas our study is composed of unrelated individuals. These observed associations are either intergenic or intronic variants suggesting that they are tagging nearby rare variants that may be causal. In most GWAS, the SNPs showing evidence of association are not usually the actual causal variants at play but rather are in LD with such variants. The failure to replicate the previously reported haplotype associations with ASD and PRKCB1 and any specific SNP associations in both PRKCB1 and CBLN1 does not invalidate the findings in this study. Over the last 5 years, considerable attention has been paid to the potential reasons why true associations may not replicate across independent data sets and genetic heterogeneity, environmental interactions, age-dependent effects, epistasis, and inadequate statistical power have all been cited as possible explanations (Chanock et al., 2007; Shriner et al., 2007; Williams et al., 2007; Greene et al., 2009).

The identification and characterization of the causal variants at play in common complex human traits/diseases is presenting to be a great challenge in human genetics, in particular as a large proportion are not in coding regions directly altering protein structure/function but rather are in non-coding regions (i.e., intronic and intergenic regions) having a likely regulatory function, such as may be the case for the *PRKCB1* intronic rs198198 SNP identified in this study. The further characterization of this and other genetic variation within the *PRKCB1* and *CBLN1* gene regions is beyond the scope of this study possibly requiring exhaustive DNA sequencing to identify all variants within the regions of association followed by a variety of detailed molecular biological analyses, like those we have recently described (Karimi et al., 2009; Kaskow et al., 2013).

## Conflict of Interest Statement

The authors declare that the research was conducted in the absence of any commercial or financial relationships that could be construed as a potential conflict of interest.

## Supplementary Material

The Supplementary Material for this article can be found online at http://www.frontiersin.org/Human_Neuroscience/10.3389/fnhum.2013.00658/abstract

Figure S1 **Histogram and Quantile–Quantile Plot of Total AQ Scores**. Histograms depict the frequency of the Total AQ observations. Q–Q plots compare the quantiles from the current study (sample quantiles) to the quantiles from a Normal distribution (theoretical quantiles).

Figure S2 **Histogram and Quantile–Quantile Plot of Social Skills Scores**. Histogram depicts the frequency of the Social Skills observations. Q–Q plots compare the quantiles from the current study (sample quantiles) to the quantiles from a Normal distribution (theoretical quantiles).

Figure S3 **Histogram and Quantile–Quantile Plot of Details/Patterns Scores**. Histogram depicts the frequency of the Details/Patterns observations. Q–Q plots compare the quantiles from the current study (sample quantiles) to the quantiles from a Normal distribution (theoretical quantiles).

Figure S4 **Histogram and Quantile–Quantile Plot of Communication/Mindreading Scores**. Histogram depicts the frequency of the Communication/Mindreading observations. Q–Q plots compare the quantiles from the current study (sample quantiles) to the quantiles from a Normal distribution (theoretical quantiles).
