# Supplementary figures and images for: Genome-Wide Association Study of Autistic-Like Traits in a General Population Study of Young Adults
Source: Front Hum Neurosci. 2013 Oct 11;7:658. doi: 10.3389/fnhum.2013.00658 (PMC3795398; doi:10.3389/fnhum.2013.00658)

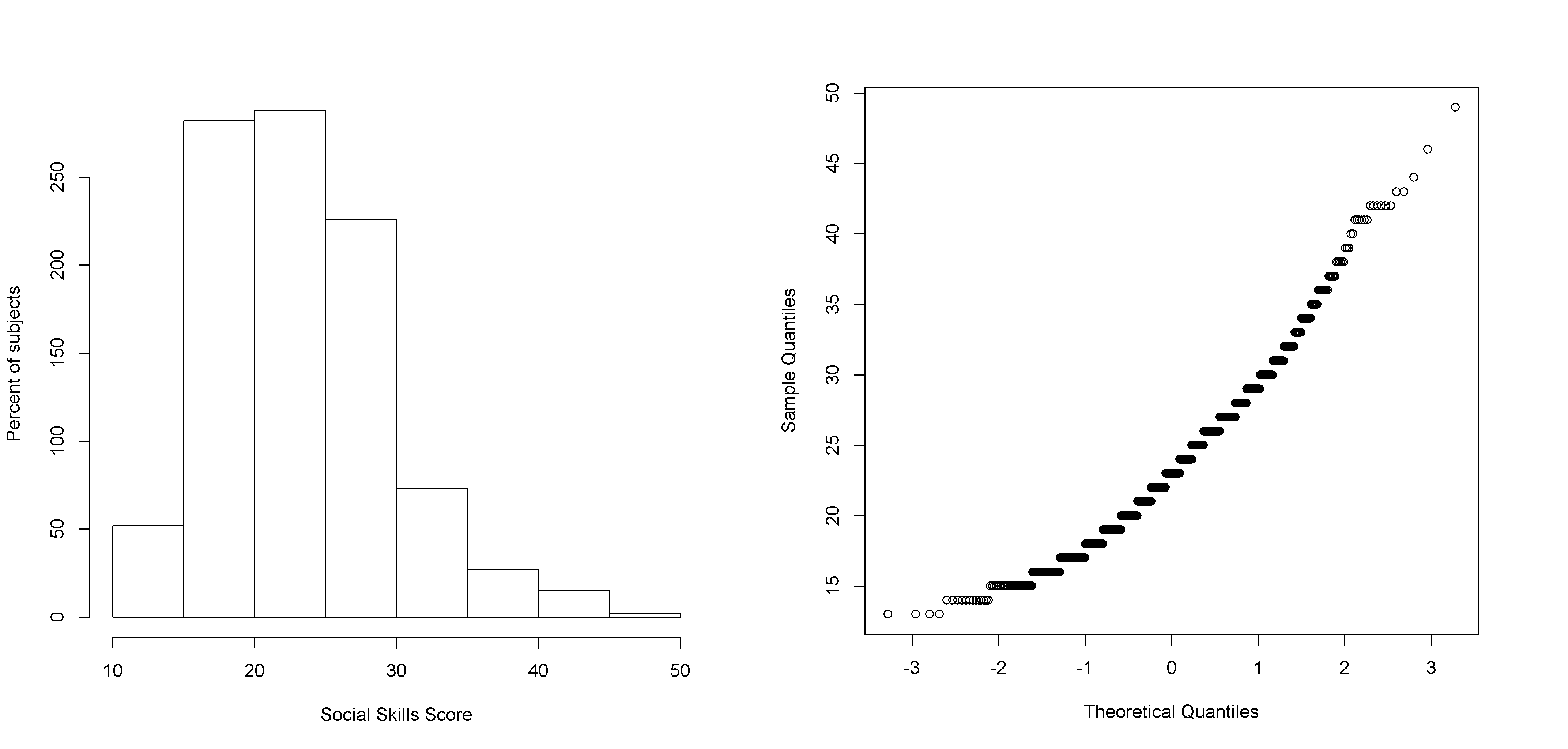

Supplement: Figure S1 — Histogram and Quantile–Quantile Plot of Total AQ Scores. Histograms depict the frequency of the Total AQ observations. Q–Q plots compare the quantiles from the current study (sample quantiles) to the quantiles from a Normal distribution (theoretical quantiles). [file 54229_Moses_Presentation1.ZIP › 54229_Moses_S2.TIF]

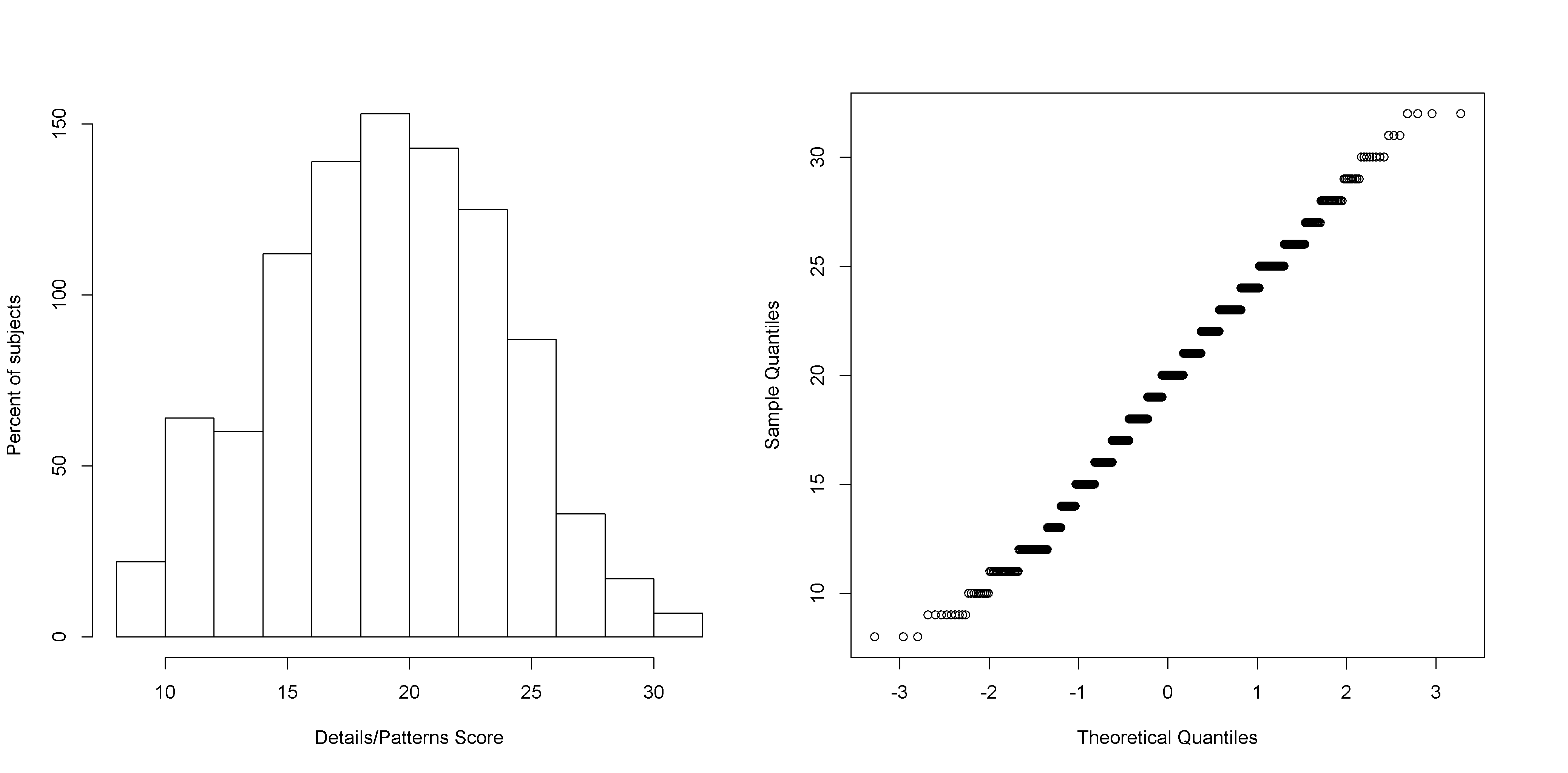

Supplement: Figure S1 — Histogram and Quantile–Quantile Plot of Total AQ Scores. Histograms depict the frequency of the Total AQ observations. Q–Q plots compare the quantiles from the current study (sample quantiles) to the quantiles from a Normal distribution (theoretical quantiles). [file 54229_Moses_Presentation1.ZIP › 54229_Moses_S3.TIF]

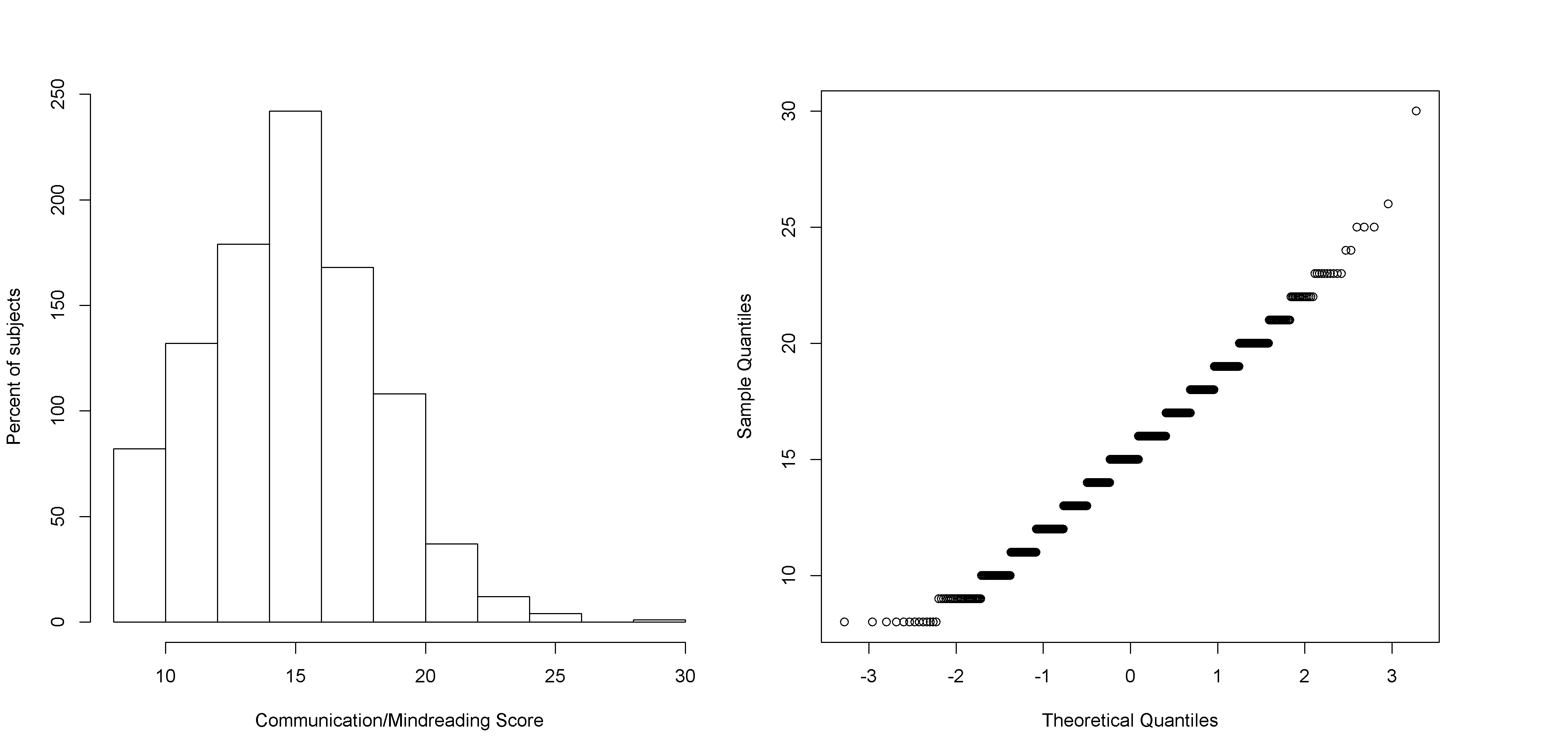

Supplement: Figure S1 — Histogram and Quantile–Quantile Plot of Total AQ Scores. Histograms depict the frequency of the Total AQ observations. Q–Q plots compare the quantiles from the current study (sample quantiles) to the quantiles from a Normal distribution (theoretical quantiles). [file 54229_Moses_Presentation1.ZIP › 54229_Moses_S4.TIF]

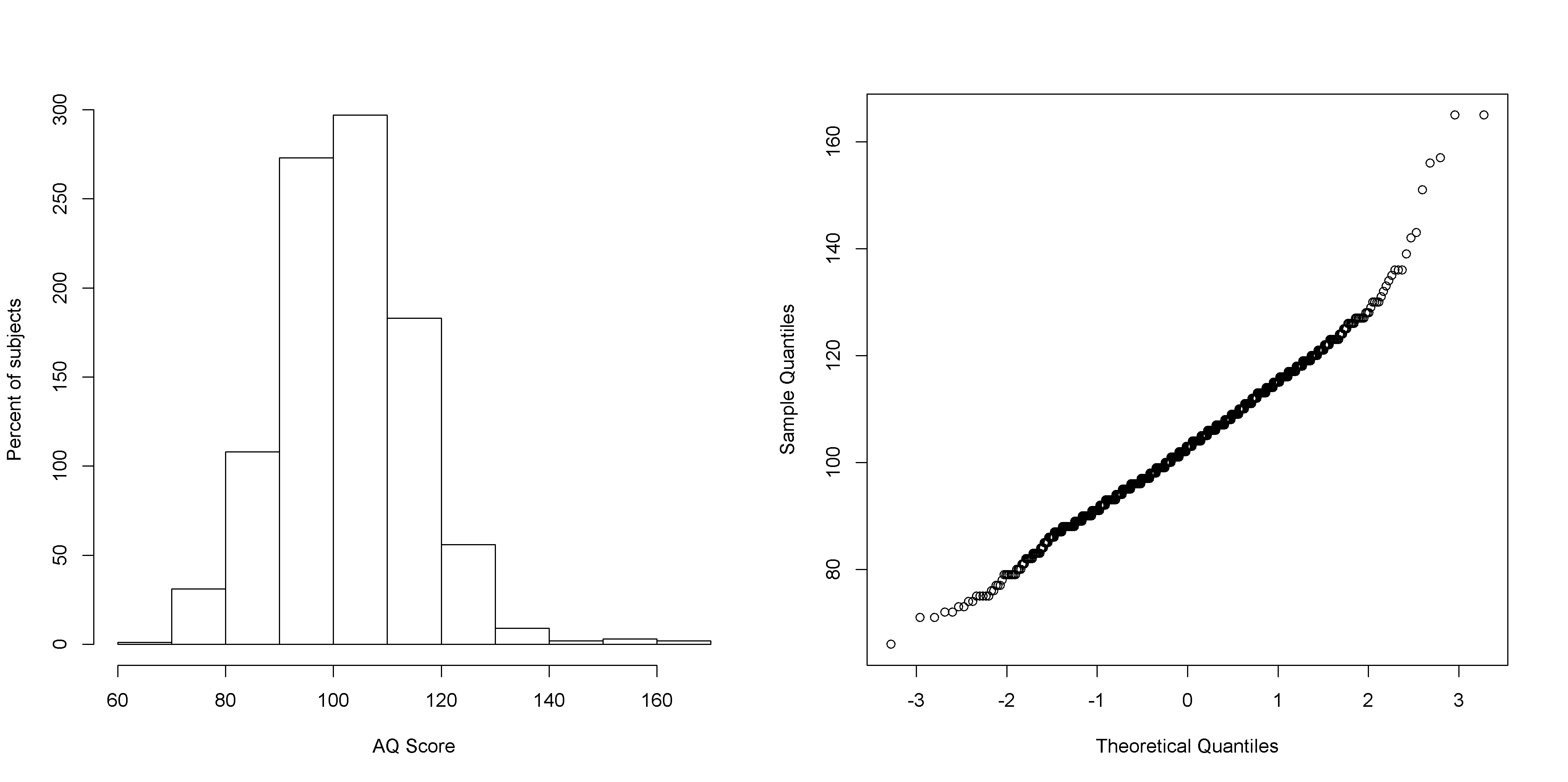

Supplement: Figure S1 — Histogram and Quantile–Quantile Plot of Total AQ Scores. Histograms depict the frequency of the Total AQ observations. Q–Q plots compare the quantiles from the current study (sample quantiles) to the quantiles from a Normal distribution (theoretical quantiles). [file 54229_Moses_Presentation1.ZIP › 54229_Moses_S1.TIF]
